# The Truth Shall Make Us Free

**Published:** 1896-04

**Authors:** J. H. Allen

**Affiliations:** Logansport, Ind.


### "THE TRUTH SHALL MAKE US FREE."

(FOURTH ARTICLE.)

J. H. ALLEN, M. D., LOGANSPORT, IND.

At the close of my last article, under the above heading, in THE HOMŒOPATHIC PHYSICIAN for January, 1896, page 18, I told you that "truth enables us to look into the mysteries of life with eyes of the soul and to put ourselves into true relationship with nature and her powers," or, in other words, it brings us into true harmony with nature's forces. In the first place, truth is an abstract thought, schic in its nature (born of its soul), a conception independent of the senses, like the word *Idea,* it is, I or the ego and Deus or God, or the Deus thought into the ego. All truth then must be born of God, and every true thought that we think must be first thought by Him as He is the aproisa of all things whether they be of the finite or the infinite. "Look at me in the eyes," said the father of Virginius to Claudius, "and tell me that she is your slave." Claudius having stared him in the face said "It is so." "Ah!" said the loving father, "you are looking through the eyes of the physical and not through the eyes of the soul," and how true that is, we cannot see the truth through our physical eyes, it is only through the eyes of the psychia that we are brought into true relationship with even an attribute of the truth, so when Hahnemann conceived of that most profound conception of his age, Similias Law, he fully appreciated the fact that it was God-given, as he so many times refers to it in his writings, and we, too, must have that higher conception of it as a science in order to fully understand the marvelous internal workings of its mighty dynamis. Seeing it in this light we cannot but exclaim from our innermost soul, as did Kepler on his discovery of the so-called harmonic laws by the theory of the elliptical orbit: "*Almighty God, I think Thy thoughts after Thee!*" This is true science, when we think, act, and follow out the dictates of omnipotence in the fulfillment of His laws.

Again, truth brings light, for it is light, and as light reveals all things in the natural universe, so truth reveals the things of the invisible, through the understanding in the true light of wisdom, for wisdom is light and is born of that true knowledge that illumineth the soul, so that he who is guided by it is led through the shadows and fogs of unbelief and doubt. Skepticism and prejudice fly away and the margin of the unknown shore constantly recedes, and the X-rays of truth penetrate the black barriers of materialism, whose walls become crystalline, and the revelation is complete. Oh! where will not truth go, it began at creation with light, and it is still the great and only true illuminator and revealer of things finite and infinite.

But, above all things, *truth* is life, for law is truth, and law applied in truth not only creates life but maintains life, and to be outside of the pale of truth is to be out of harmony, and to be out of harmony with those great gravitational forces in nature that govern all true harmonics is to disturb true biological law, and to disturb biological law is to hobnob with death. Now, through this great light that the truth in Homœopathy reveals to us, we have found that the action of these subversive forces found to produce disease can be arrested in their action upon the life forces, by other subversive forces known as potentized medicines, which, being applied through the law, we return to health, and all the normal functions of the body are again restored by virtue of their true harmonic relationship and their co-existing similarity of action, having their parts, forms, actions, and potencies (if we may be allowed to use such terms in this connection) in proper accordance with each other, so that where discord was now harmony exists, supplementing it, showing that everything that draws its origin from truth manifests itself in all the true relationships that evolve out of order, for if anything evolves not out of truth it must be a discord, even its very existence must be full of discord and deformity. "O judgment! thou hast fled to brutish beasts and men have lost their reason" because they are not led by the unerring hand of truth, therefore they are out of the order of harmonics, and under the serfdom of the subversive powers, and Pandora-like, through our ignorance, we open the Prometheus-bound box that was sealed by the seal of true harmonics, and out cometh nothing but complexity and confusion. "God exists," says Emerson, "and there is a soul at the centre of nature and over the will of every man, so we cannot wrong the universe." Listen and you will hear the voice of truth calling unto you out of the babel of world's voices, saying, "Come this way! Come this way!" and if we place ourselves in the centre of its full stream of power it will push us on in the right direction without any effort on our part, propelling us on into the light of truth and power. To-day the great truth of Homœopathy is knocking at your door. Why do you not let him in? You are as worthy as the thousands that go by. Let him in and be a king. What is the use of fighting longer the eternal principles of the universe? Is it not better to be able to take hold of the levers of the mighty dynamis of nature's forces than to be crushed under their wheels, if there are forces and powers that inhibit or control other powers and forces, and especially the disturbed life forces? Let us use them through law, and not as bats that knock out their lives against the night lamps because their eyes are not fitted to receive the light. We can, by our knowledge of the true nature of forces, insulate them so as to control them with a touch button, when, if they are not under our control, they may turn upon us and destroy us. If the proper potency of the force taken from Belladonna is given in a case of scarlet fever, chosen through the law of similia, every tissue in the body is constrained to offer testimony that you have complied with law, and as the light hates the darkness and is ever transforming it into light, so the action of the proper remedy is constantly changing sickness into health. One is law subversively or negatively in action, and the other is law positively or harmonically in action, and when the powers of truth, which are life, are in the positive action, then the opposite to truth, or death and dissolution, must become subordinate to them, and a complete subordination to similia is health. In the first place, we must subordinate ourselves to law, and by virtue of that subordination cometh the simillimum under which not only the life forces must become subordinate, but the subversive forces themselves must become subordinate. Thus disease is the life force + the disturbing subversive power and health is the life forces + the subversive force + similia, which is truth itself, or restored harmony.

